# A Novel Method for Achieving Metacarpophalangeal Joint Stability in Cases of Unstable Thumb Duplication: A Report of Two Cases

**DOI:** 10.7759/cureus.78224

**Published:** 2025-01-30

**Authors:** Kaoru Sasaki, Junya Oshima, Yukiko Aihara, Yoichiro Shibuya, Mitsuru Sekido

**Affiliations:** 1 Department of Plastic, Reconstructive, and Hand Surgery, University of Tsukuba, Tsukuba, JPN

**Keywords:** duplication, ligament, polydactyly, stability, tendon, thumb

## Abstract

Two factors contribute to the stability of the thumb: static stability provided by the bones, joints, and ligaments, and dynamic stability provided by the muscles. Proximal types of polydactyly, such as Wassel types Ⅴ and Ⅵ, are often associated with thenar dysplasia, which is thought to lack sufficient dynamic stability. Therefore, in cases of proximal polydactyly where instability at the metacarpophalangeal (MCP) joint is severe, static stability becomes important. No reports have been published on ligament reconstruction during primary collateral ligament reconstruction for thumb polydactyly, a pediatric congenital difference. We achieved good midterm results with the reconstruction of the ulnar collateral ligament of the MCP joint using the spare flexor pollicis longus tendon for MCP joint instability. This procedure may be an option for MCP joint stabilization in patients with primary surgery presenting MCP joint ulnar instability.

## Introduction

Stability of the reconstructed thumb is important in the treatment of polydactyly [1-4]. However, cases of congenital hypoplasia or deficiency of the articular collateral ligaments occasionally occur, leading to problematic postoperative instability of the reconstructed thumb.

Two factors contribute to the stability of the thumb: static stability provided by the bones, joints, and ligaments, and dynamic stability provided by the muscles. Proximal types of polydactyly, such as Wassel types Ⅴ and Ⅵ, are often associated with thenar dysplasia [5,6], in which the dynamic stability is thought to be insufficient compared to that of the distal type. Therefore, in proximal polydactyly, where instability at the metacarpophalangeal (MCP) joint is severe, static stability is crucial.

Although ligament reconstruction is the standard treatment for joint instability due to traumatic ulnar ligament rupture of the MCP joint, no reports have been published on primary ligament reconstruction for thumb polydactyly, a pediatric congenital anomaly. Additionally, only a few reports exist on the use of autologous tissue to stabilize the joint for thumb polydactyly [2,3,7]. Here, we describe two cases of proximal type thumb polydactyly in which collateral ligament reconstruction was performed using the tendon and articular capsule of the resected digit.

This article was previously presented as a meeting abstract at the 65th annual meeting of the Japanese Society for Surgery of the Hand on April 15, 2022.

## Case presentation

Case 1

**Figure 1:**
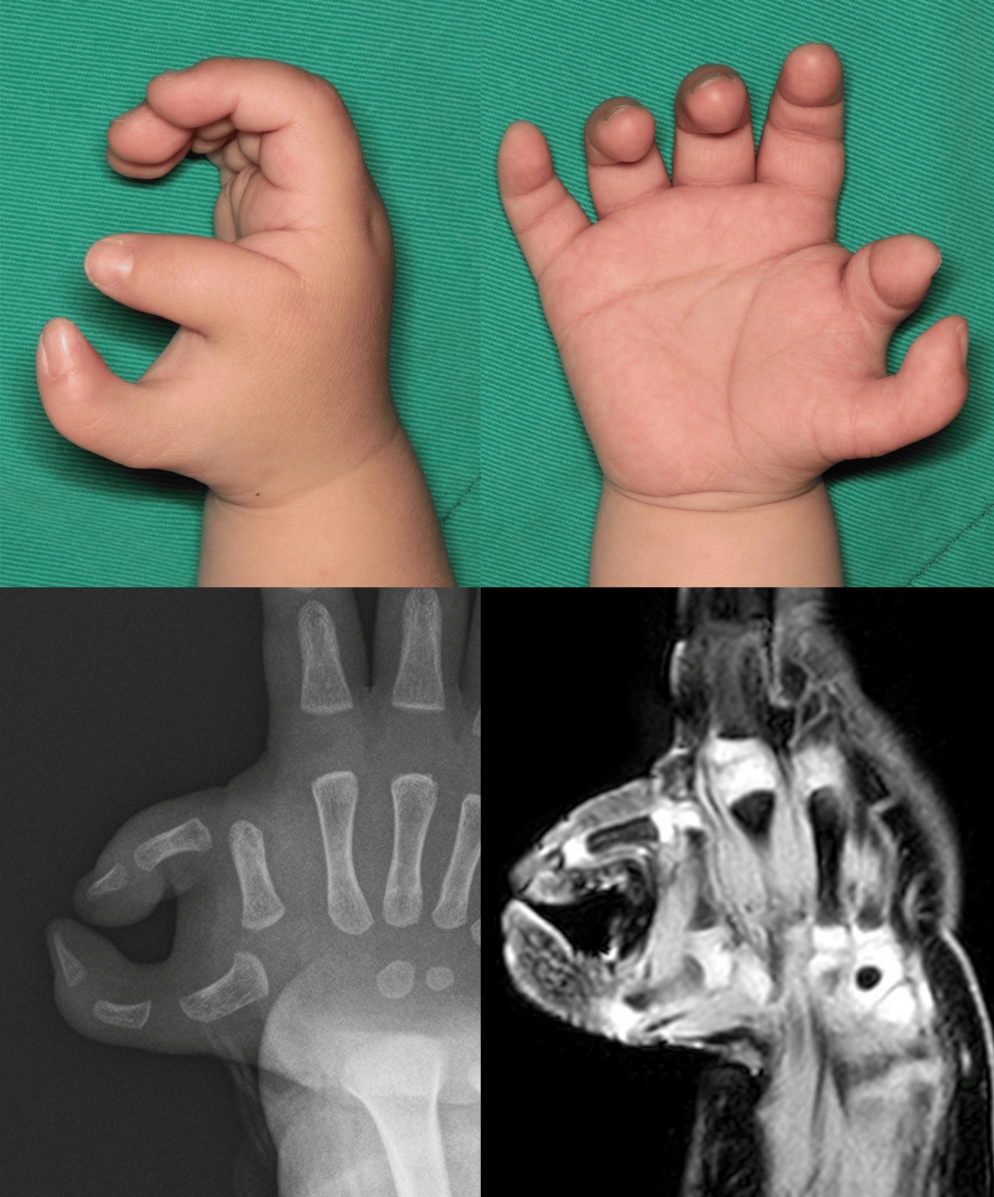
Preoperative findings. A one-year-old boy with Wassel type VI polydactyly. Both the radial and ulnar digits showed severe lateral instability at the metacarpophalangeal (MCP) joint. Preoperative MRI confirmed that the MCP joint structure of the ulnar digit was intact.

A one-year-old boy with a Wassel type VI duplicate thumb showed severe MCP joint instability of both thumbs. An MCP joint stress test under X-ray demonstrated -10 degrees ulnar deviation and 100 degrees radial deviation of the ulnar thumb, and 70 degrees radial deviation and 42 degrees ulnar deviation of the radial thumb (Table 1). The ulnar thumb was superior in terms of shape and size. Preoperative MRI confirmed that the MCP joint structure of the ulnar digit was intact (Figure 1).

**Table 1:** Patient characteristics. Metacarpophalangeal (MP) joint stability improved, and phalangeal growth disturbances were negligible.

	Stress test				The ratio of the affected phalanx length to the healthy phalanx length				Duration for follow-up	JSSH score
											Pre-ope		Post-ope		Pre-ope		Post-ope	
	Ulnar deviation	Radial deviation	Ulnar deviation	Radial deviation	Proximal phalanx	Metacarpal bone	Proximal phalanx	Metacarpal bone		
											Case 1	-10	100	17	20	0.96	0.87	1.02	0.97	5 years	18
	Case 2	0	65	12	20	1.04	0.92	0.97			0.96	5 years 11 months	19

**Figure 2:**
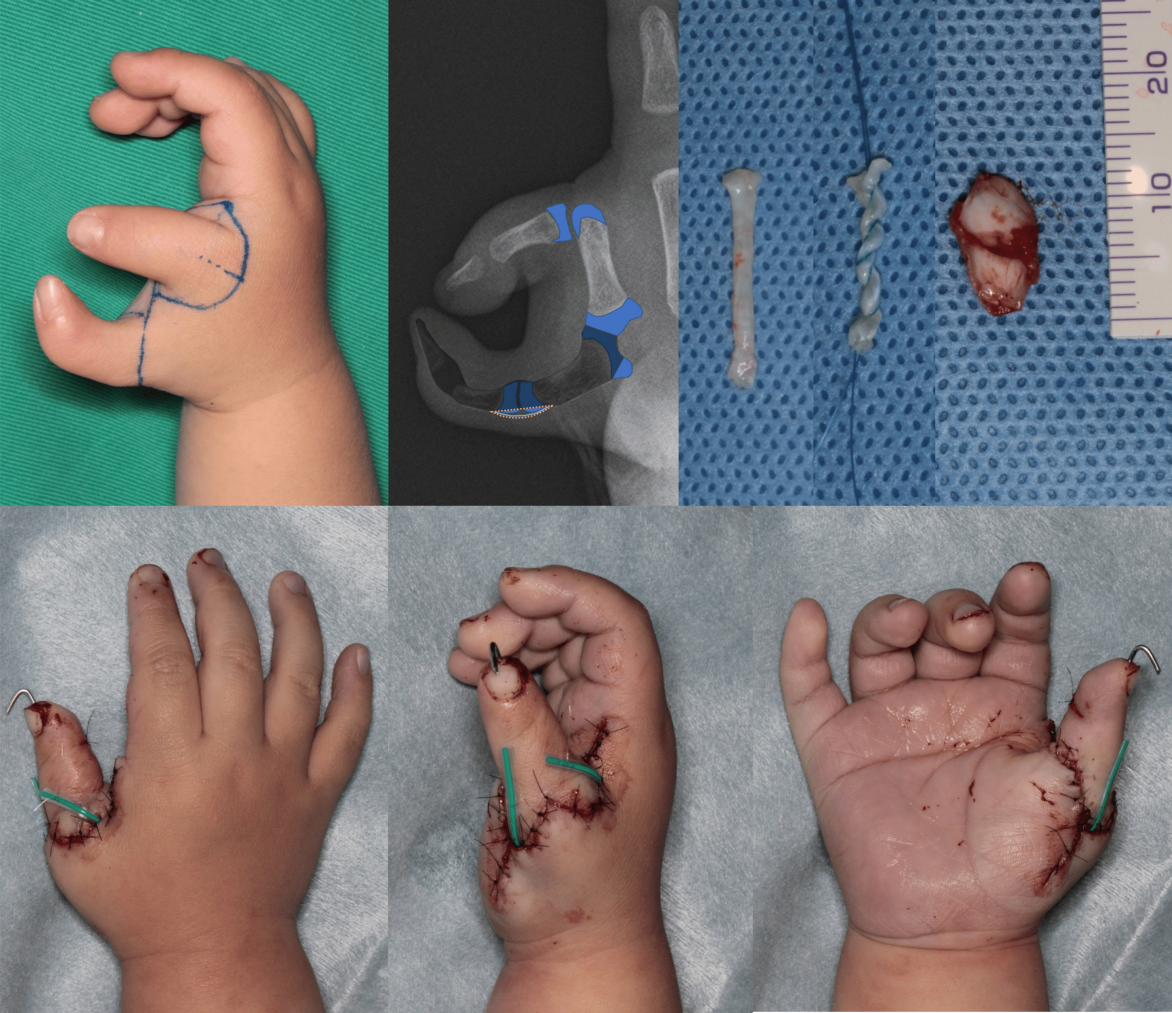
Intraoperative findings of case 1. The flexor pollicis longus (FPL) tendon and articular capsule were harvested as grafts. The articular cartilage contained within the articular capsule was removed. At the end of the surgery, the reconstructed thumb was in a good position.

At surgery, the skin incision was designed to widen the first interdigital space and facilitate thumb opposition (Figure 2). In the carpometacarpal joint, the radial side of the metacarpal base, including the insertion of the abductor pollicis longus, was preserved, and the radial digit was resected. The ulnar digit and remnants of the radial metacarpal base, including the insertion of the abductor pollicis longus, were sutured and fixed at the cartilage to form the carpometacarpal joint.

The flexor pollicis longus (FPL) tendon and MCP articular capsule from the excised radial digit were harvested as graft material (Figure 2). To correct the deviation of the FPL insertion at the distal phalanx, the FPL tendon was relocated and rebalanced. The extensor pollicis longus tendon of each finger was connected by an extensor hood-like membranous structure.

**Figure 3:**
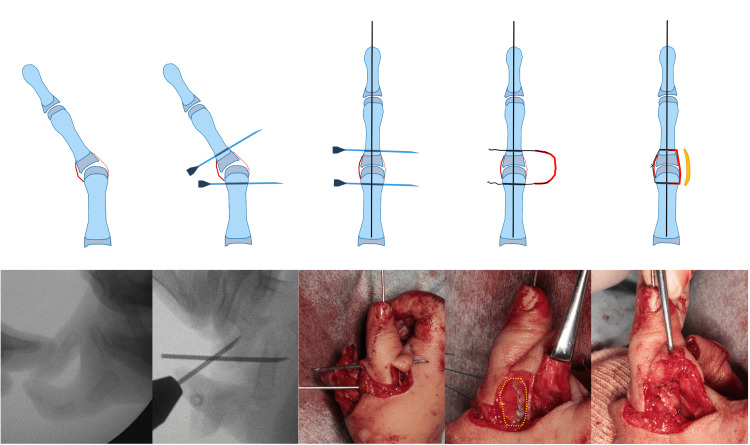
Ligament reconstruction procedure. First, a bony foramen was created in the basal and metacarpal bones using a needle. Next, K-wire fixation was performed to ensure good phalangeal alignment. The threads at both ends of the flexor pollicis longus (FPL) tendon were pulled out and then ligated on the radial side. The harvested articular capsule was sutured to the dorsal capsule and reconstructed ligament to prevent slippage in the palmar direction during metacarpophalangeal (MCP) joint flexion. The sutured area of the articular capsule is indicated by a yellow dotted circle.

**Figure 4:**
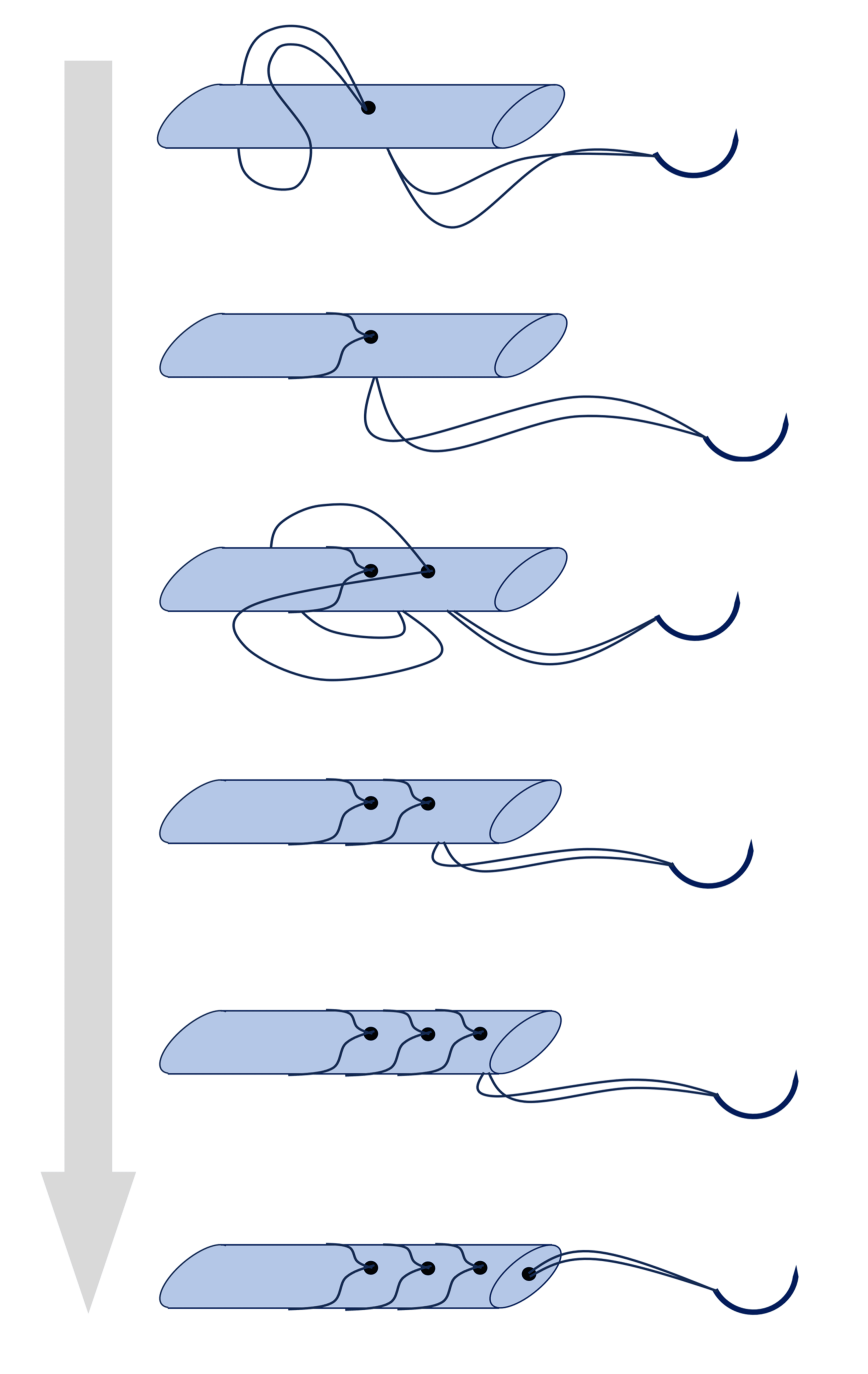
Suturing technique for the transplanted tendon. Both ends of the harvested flexor pollicis longus (FPL) tendon were sutured using the looped whipstitch technique with nylon loop thread.

The following procedure was performed for ligament formation at the MCP joint (Figure 3). To avoid injury to the epiphysis, 18G needles were used to drill and penetrate perpendicularly into the bony part of the digit axis. When the needles inserted into each metacarpal and basal phalanx were parallel and the finger axis alignment was correct, a 0.7-mm K-wire was inserted longitudinally. Both ends of the harvested FPL tendon were sutured using the looped whipstitch technique [8] with 4-0 nylon loop thread (Figure 4) and guided to the radial side of the MCP joint through the inserted needles. The pulled-out traction thread was ligated on the radial side as necessary to eliminate lateral instability of the MCP joint, and the ulnar collateral ligament of the MCP joint was reconstructed.

The harvested articular capsule was sutured to the dorsal capsule and the reconstructed ligament to prevent slippage in the palmar direction during MCP joint flexion. Regarding the thenar muscles, preoperative MRI showed that the opponens pollicis (OP), abductor pollicis brevis (APB), and flexor pollicis brevis (FPB) appeared to insert at the radial proximal phalanx, while the adductor pollicis (AP) appeared to insert at the ulnar phalanx. The thenar muscles attached to the resected digit were elevated along with the periosteum, including the insertion, and sutured to the dorsal ulnar periosteum of the basal phalanx (Figure 2).

**Figure 5:**
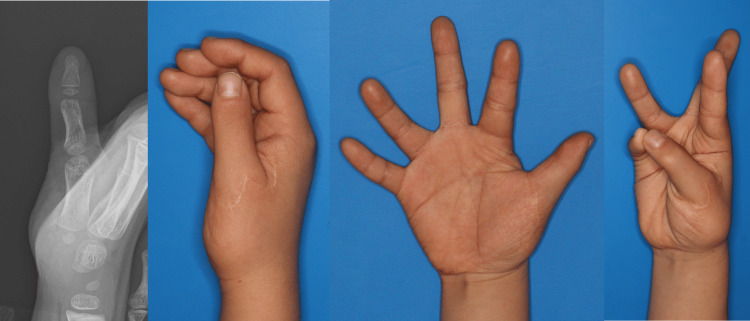
Five years after surgery in case 1. The metacarpophalangeal (MCP) joint was stable, and the affected hand was used as the dominant hand.

The K-wire was removed four weeks postoperatively. The patient had no postoperative complications. Five years after the surgery, a stress test of the MCP joint showed no instability at 17 degrees of ulnar deviation or at 20 degrees of radial deviation. The patient uses the affected hand as his dominant hand (Figure 5). Phalangeal growth was assessed on X-ray. The lengths of the basal phalanx and metacarpal were measured from the distal end to the center of the MCP joint and from the center of the MCP joint to the proximal end, respectively. The ratio of the length of the affected phalanx to that of the healthy phalanx (length of affected phalanx/length of healthy phalanx) was calculated, and both the basal and metacarpal bones showed a mild increase, indicating no bone growth disturbance. Final assessments were made according to the Japanese Society for Surgery of the Hand (JSSH) evaluation. The JSSH evaluation score was 18/20 (good) (Table 1) [9].

Case 2

**Figure 6:**
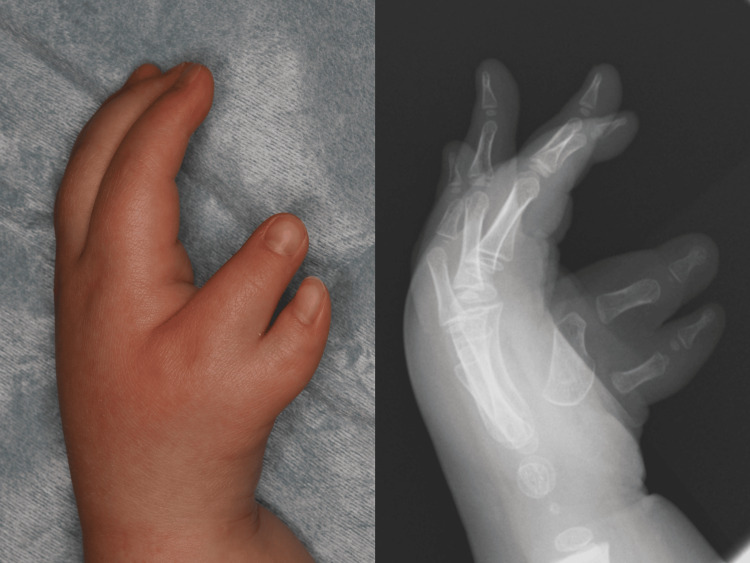
Preoperative findings of case 2. A three-year-old boy with Wassel type V polydactyly. The metacarpophalangeal (MCP) joint instability of the radial digit was severe.

A three-year-old boy with Wassel type V polydactyly underwent surgery for thumb polydactyly after radical surgery for concomitant pulmonary atresia. The radial finger was hypoplastic, and the MCP joint of the predominantly large ulnar finger showed severe lateral instability (Figure 6). An ulnar MCP joint stress test performed under X-ray showed that the radial digit had 0 degrees of ulnar deviation and 65 degrees of radial deviation (Table 1). In addition, malalignment of the first metacarpal bone and narrowing of the first interphalangeal space were observed.

**Figure 7:**
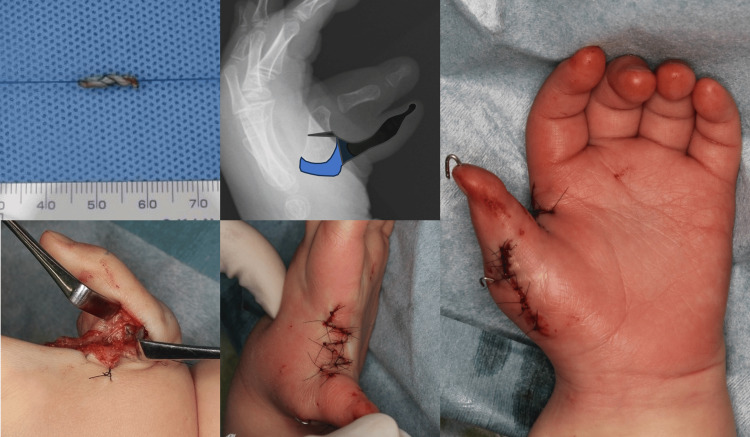
Intraoperative findings of case 2. The ligament reconstruction was performed using the flexor pollicis longus (FPL) tendon. The interdigital space was enlarged.

The surgical findings were as follows (Figure 7). A spindle-shaped skin incision was performed at the base of the excised digit. Five-flap plasty was also performed to widen the interdigital space. The FPL tendon was Y-shaped. And the radial side was harvested as the graft. The metacarpal was fixed with a 1-mm K-wire and a 0.7-mm K-wire in the internal rotation position to achieve an opposing position after a wedge-shaped rotational osteotomy. Collateral ligament reconstruction was performed following the same procedure as that in case 1, with the exception that 3-0, instead of 4-0, nylon loop sutures were used. The APB was elevated from the radial digit and suture-fixed to the extensor hood at the base of the ulnar digit with 4-0 absorbable monofilament thread. In case 2, unlike in case 1, no signs of slippage of the reconstructed ligament were observed intraoperatively; therefore, additional transplant of the articular capsule was not performed.

**Figure 8:**
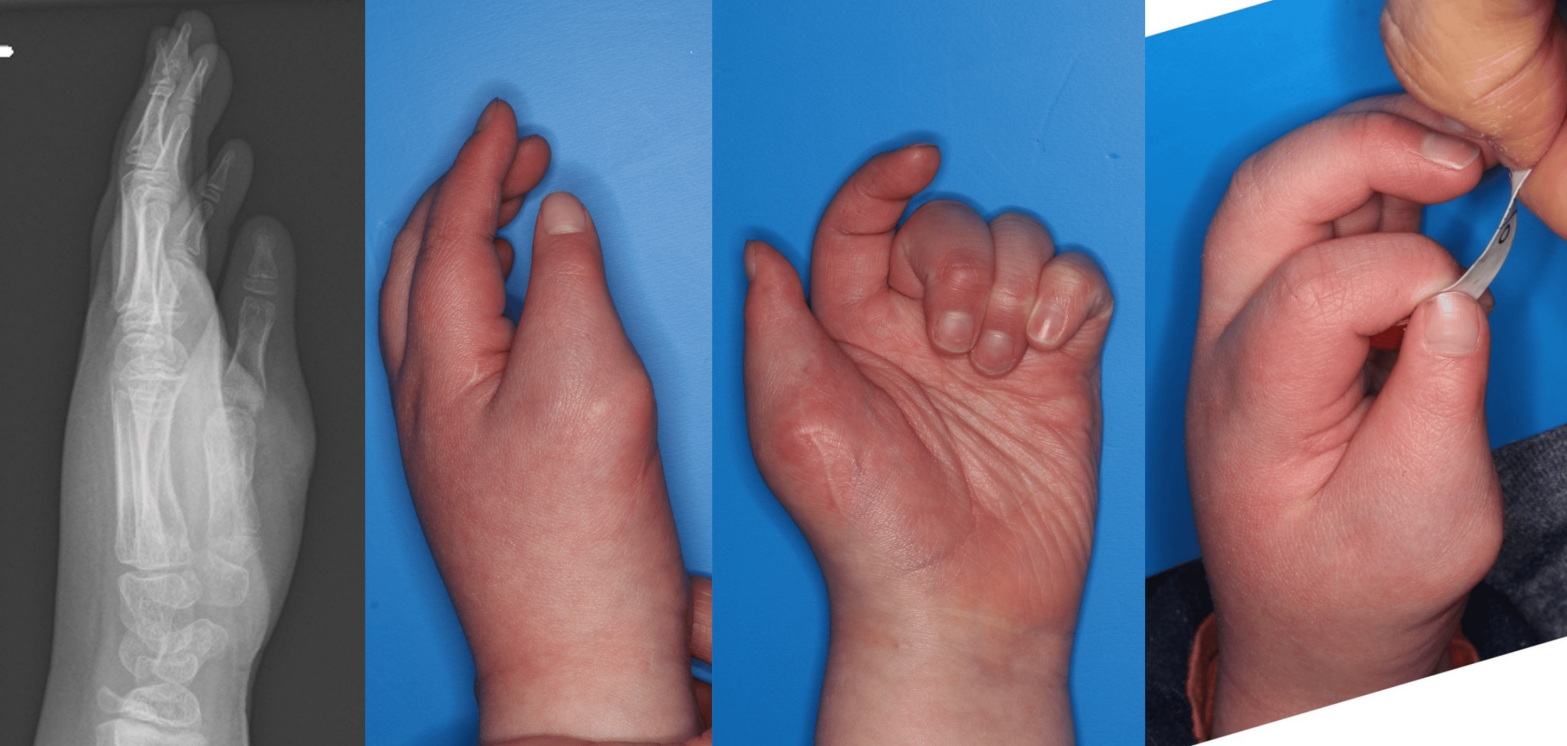
Five years and 11 months after surgery in case 2. The metacarpophalangeal (MCP) joint was stable, and the affected hand was used as the dominant hand.

The K-wire was removed six weeks postoperatively. The postoperative period passed without complications. At five years and 11 months postoperatively, stress testing showed 12 degrees of ulnar deviation and 20 degrees of radial deviation, with no instability in the MCP joint (Figure 8). The pinch function was satisfactory. The affected hand was used as the dominant hand. The ratio of the affected phalanx length to the healthy phalanx length was slightly increased in the osteotomized metacarpal but slightly decreased, from 1.04 to 0.97, in the basal phalanx. The radiographic joint gap was narrowed, resulting in mild degenerative changes. The JSSH evaluation score was 19/20 (good) (Table 1) [9].

## Discussion

Unlike trauma injuries, congenital hand differences are often treated conservatively and defensively due to the need to avoid growth retardation and the expectation of adaptability in children. In cases of duplicate thumbs, we sometimes encounter patients with radial deviation deformity and MCP joint instability over the long term. However, static stabilization of joints with severe preoperative instability is rarely performed during the primary surgery, and muscle transfer and rebalancing are often performed for dynamic stabilization [10]. In cases of residual instability, joint stabilization procedures, including the use of the articular capsule, may be performed at a later date. However, MCP joint instability in polydactyly may affect both the acquisition of hand functionality and bone development during the growth process, making it ideal to address this issue with single-stage surgery rather than a two-stage approach. The primary collateral ligament reconstruction reported in this study is an effective method for stabilizing the MCP joint with minimal invasiveness by making optimal use of tissues that would otherwise be discarded in congenital hand anomalies.

We consider three conditions as indications for primary collateral ligament reconstruction. First, the case should involve proximal bifurcation types, such as Wassel types V and VI, with associated thenar dysplasia. This is because, in Wassel types III and IV, the thenar muscles are better developed than in types V and VI, providing sufficient dynamic stability to compensate for static instability. Additionally, distal bifurcation types are less likely to exhibit ligament hypoplasia-related MCP joint instability than proximal types. Second, there should be no instability or deviation of the IP joint in the preserved digit. For IP joint deviation, tendon relocation and rebalancing using the FPL are effective; therefore, careful consideration is required when using the FPL for ligament reconstruction. If both the IP and the MCP joints exhibit instability, it may be preferable to prioritize FPL usage for tendon relocation and rebalancing. In such cases, MCP joint stabilization can be managed using a transplant of the collateral ligament and articular capsule harvested from the resected digit [2]. Additionally, joint surface angle adjustment through osteotomy may also be effective. Since dynamic stability achieved through tendon relocation and balancing is crucial in the long term, the best candidates for this procedure are those not requiring the FPL of the resected digit for tendon relocation. Third, the MCP joint instability should exceed 40 degrees, with predominant deviation on one side. According to the JSSH evaluation score, MCP joints with instability of 40 degrees or less are considered stable, while those with instability of 60 degrees or more are classified as severely unstable. Therefore, we believe this method is indicated for cases with MCP joint instability exceeding 40 degrees and predominantly unilateral instability due to ligament hypoplasia.

Regarding the technique of ligating the pull-out sutures to each other on the radial side of the MCP joint, long-term follow-up of its effect on joint growth is required. In primary surgical ligament reconstruction, the possibility of long-term ligament laxity and bone growth disturbance must be considered. In our midterm outcome, no bone growth disturbance was observed in either case, and good function was achieved; however, in case 2, the joint space had become narrower and degenerative. The reasons for this are that the 3-0 nylon used in case 2 was too strong for the growing joint and the excessive pressure affected the joint over time. In summary, the disadvantage of this method is its potential to cause osteoarthritis. However, considering that conservative treatment could have resulted in joint fixation after growth, this method may offer the advantage of achieving a stable finger after primary surgery without growth disturbances or pain, as well as promoting hand functionality, which may outweigh its disadvantages. We consider the most significant advantage of this method to be the effective utilization of the resected tissue. Whilst various materials are available for ligament reconstruction, given the current lack of clarity regarding the long-term outcomes of primary collateral reconstruction, it is preferable to avoid using tissues from donor sites intended for secondary reconstruction or artificial materials that may impact growth. As mentioned earlier, the nylon thread used in this method is also an artificial material. In case 2, the joint appeared to have been affected by the nylon thread, indicating that the use of nylon in this method is an area for improvement. The development of sutures with sufficient tensile strength and an appropriate absorption period is highly anticipated.

The fact that the position of the collateral ligament to be reconstructed in this method differs from the original anatomical location also warrants discussion. To avoid damage to the epiphysis, a tunnel was prepared in a distant bony part for the passage of the transplanted ligament. This resulted in the ends of the ligaments being located farther from the joint than their anatomical locations. The caveat of this method is that depending on the angle of the joint, the grafted ligament may slip dorsally or palmarly beyond the bony head.

Both static stability and dynamic stability are important for thumb reconstruction. This method is revolutionary in that it allows for static stabilization of the MCP joint at the primary surgery. Both patients were able to obtain stable MCP joints in the medium term, and the fact that the affected hand became the dominant hand in both cases suggests that the hand in which this technique was performed was easy for the patient to use. For these patients with severe instability of the MCP joint, the early stabilization of the MCP joint improved the overall function of the hand and seemed to be a superior method to that of two-stage surgery.

This method offers advantages as it effectively utilizes discarded tissue, minimizing waste. Additionally, it shows potential for application in congenital anomalies beyond polydactyly of the thumb. However, this approach requires advanced technical skills and high-quality medical equipment, thus limiting its usage to geographical regions with sufficient numbers of hand and plastic surgeons. Consequently, its implementation may face challenges in rural or underserved regions with limited access to specialized care [11].

In terms of its limitations, this is a report of only two cases, and further larger, long-term studies are needed to evaluate hand function, joint instability, finger length, and arthritic changes.

## Conclusions

We achieved good midterm results with the reconstruction of the ulnar collateral ligament of the MCP joint using the spare FPL tendon in two cases of MCP joint ulnar instability. This procedure shows promise as an option for MCP joint stabilization during primary surgery.
